# Strategic test-day recording regimes to estimate lactation yield in tropical dairy animals

**DOI:** 10.1186/s12711-014-0078-0

**Published:** 2014-12-02

**Authors:** David M McGill, Peter C Thomson, Herman A Mulder, Jan J Lievaart

**Affiliations:** Graham Centre for Agricultural Innovation, Locked Bag 588, Wagga Wagga, NSW 2678 Australia; School of Animal and Veterinary Sciences, Charles Sturt University, Wagga Wagga, NSW 2650 Australia; Faculty of Veterinary Science, The University of Sydney, Camden, NSW 2570 Australia; Animal Breeding and Genomics Centre, Wageningen University, PO Box 338, 6700 AH Wageningen, The Netherlands

## Abstract

**Background:**

In developing dairy sectors, genetic improvement programs have limited resources and recording of herds is minimal. This study evaluated different methods to estimate lactation yield and sampling schedules with fewer test-day records per lactation to determine recording regimes that (1) estimate lactation yield with a minimal impact on the accuracy of selection and (2) optimise the available resources.

**Methods:**

Using Sahiwal cattle as a tropical dairy breed example, weekly milk records from 464 cows were used in a simulation study to generate different shaped lactation curves. The daily milk yields from these simulated lactation curves were subset to equally spaced (weekly, monthly and quarterly) and unequally spaced (with four, five or six records per lactation) test-day intervals. Lactation yield estimates were calculated from these subsets using two methods: the test-interval method and Wood’s (Nature 216:164-165, 1967) lactation curve model. Using the resulting lactation yields, breeding values were predicted and comparisons were made between the sampling regimes and estimation methods.

**Results:**

The results show that, based on the mean square error of prediction, use of Wood’s lactation curve model to estimate total yield was more accurate than use of the test-interval method. However, the differences in the ranking of animals were small, i.e. a 1 to 5% difference in accuracy. Comparisons between the different test-day sampling regimes showed that, with the same number of records per lactation (for example, quarterly and four test-days), strategically timed test-days can result in more accurate estimates of lactation yield than test-days at equal intervals.

**Conclusions:**

An important outcome of these results is that combining Wood’s model for lactation yield estimation and as few as four, five or six strategically placed test-day records can produce estimates of lactation yield that are comparable with estimates based on monthly test-day records using the test-interval method. Furthermore, calculations show that although using fewer test-days results in a decrease in the accuracy of selection, it does provide an opportunity to progeny-test more sires. Thus, using strategically timed test-days and Wood’s model to estimate lactation yield, can lead to a more efficient use of the allocated resources.

**Electronic supplementary material:**

The online version of this article (doi:10.1186/s12711-014-0078-0) contains supplementary material, which is available to authorized users.

## Background

Breed improvement and selection in dairy systems of developing countries is a challenge because field conditions are restricted by limited resources and infrequent milk recording. In these situations, frequent test-day (TD) recording throughout the entire lactation for genetic evaluation purposes is difficult and impractical [1]. This lack of data availability highlights the need to optimise the contribution of each collected record to the genetic evaluation process [2]. Therefore, in such situations, there is a need to develop an efficient TD sampling regime and subsequent genetic evaluation system that optimises selection outcomes given the current resources for TD recording.

In dairy systems, genetic test-day models (TDM) provide a solution to the lack of data since they can effectively use fewer records [3-5]. However, TDM require accurate estimates of many genetic parameters calculated from large datasets [6,7], which are difficult to obtain in developing country scenarios and hence can cause inaccurate results [3,8]. Therefore, an approach in which lactation yield is first estimated and subsequently used for breeding value estimation may be more appropriate.

There are numerous methods to estimate lactation yield based on TD records. The test-interval method (TIM) is the reference method to calculate lactation yield [9] and is particularly useful in developing dairy sectors. Other methods involve the use of mathematical lactation models to predict milk yield. TD data from Sahiwal cattle, a tropical dairy breed from Pakistan [10], have been used in a number of studies that compare different lactation models. These studies indicated that suitable models include the inverse polynomial function proposed by Nelder [11], the incomplete gamma-type function proposed by Wood [12] and the Wilmink model [13-16]. Various studies have investigated which of each these models is the most appropriate in different tropical or sub-tropical conditions [11,17-20]. Although some results of these studies are conflicting, it is clear that the most suitable model is the Wood model because of its ability to fit different shaped curves and its relative ease to describe characteristics of the lactation curve [20-22]. Furthermore, we recently reported that the Wood model is more robust than others when fitting lactation curves to infrequent and irregular test-day sampling regimes (TDSR), which are common in developing country scenarios [23]. For these reasons, the Wood model was used for data modelling and simulation of datasets for this study, although it is expected that other models of similar complexity would give similar results.

Previous studies have clearly shown that as milk recording frequency decreases, the accuracy of the lactation yield estimates also decreases [24-27]. Despite this, it is possible to record milk yield monthly or even just four or five times throughout a lactation and still estimate lactation yields sufficiently accurately to rank cows for selection based on milk production [25-27]. Some studies considered using unequally spaced sampling regimes with more TD around the peak of lactation or timed according to the visits of an AI (artificial insemination) technician. These studies found that, although the lactation yield estimates were less accurate when TD were unequally spaced [28], they did provide an opportunity to assess more bulls with the same resources while maintaining the reliability of the resulting sires’ estimated breeding values (EBV) [27]. What has not been directly considered is whether as few as four, five, or six TD strategically timed throughout lactation would have an effect on the accuracy of EBV for lactation yield.

Therefore, the aim of this study was to investigate whether using fewer, but more strategic TD sampling approaches, and the Wood lactation model would improve the accuracy of lactation yield estimates and EBV for milk production. Milk records from Sahiwal cattle were used to simulate lactation curves as an example of the production in tropical dairy breeds. The simulated data were then used to compare different TDSR with only four, five or six sampling days within one lactation. Then, comparisons of EBV between sampling and estimation methods were assessed to determine the most efficient approach to estimate breeding values given the resources available.

## Methods

### Raw data

Sahiwal cattle were used as an example of a typical tropical dairy breed. Sahiwal lactation records collected between 2005 and 2010 from the Livestock Production Research Institute (LPRI), Bahadurnagar Okara, Pakistan, were used. The raw data consisted of 839 lactations with weekly TD records from 464 dams from 82 sires, with an average of 5.65 daughters per sire. The dams ranged in age from three to over ten years, with approximately 25% of lactations from cows in their first lactation, 45% from the second to fourth lactation, and the remaining 30% from the fifth lactation and above. Using these TD records, Wood’s [12] lactation curve model was fitted to each lactation. This model is defined as follows in its original nonlinear form (1) and its linear form (2):

1 $$ W(t)=a{t}^b{e}^{-ct} $$

2 $$ W(t)= \exp \left(k+b \ln t-ct\right), $$

where *W*(*t*) is the model-based lactation yield at time *t* (days in milk (DIM)), *k =* ln*a*, and where *k*, *b*, and *c* specify the shape of the lactation curve.

### Analysis of raw data

To obtain estimates of the various components of the Wood model for the simulation study, the raw Sahiwal TD records were fitted using a nonlinear mixed model. This was done by the nlme() function of R Version 3.0.2 [29], using the methods documented in Pinheiro et al. [30]. In this case the model fitted was:

3 $$ {y}_{it}= \exp \left({k}_i+{b}_i \ln t-{c}_it\right)+{\varepsilon}_{it}, $$

where ${\varepsilon}_{it}\sim N\left(0,{\sigma}_{\varepsilon}^2\right)$, *i* indexes the particular cow, with *i* = 1, 2, …, *n* and *n* is the number of cows in the dataset.

The linear form (3) of the Wood model was used here because of its closer approximation of parameters (*k*_*i*_, *b*_*i*_, *c*_*i*_) to a multivariate normal distribution [22], as required for the model assumptions to be met.

The Wood model can be developed further as a nonlinear mixed model with additive (linear) sub-models for *k*_*i*_, *b*_*i*_ and *c*_*i*_ such that:

4 $$ \begin{array}{c}\hfill {k}_i=k+{k}_{MOC}+{k}_{Year}+{k}_{Age}+{K}_{i.G}+{K}_{i.E}\hfill \\ {}\hfill {b}_i=b+{b}_{MOC}+{b}_{Year}+{b}_{Age}+{B_i}_{.G}+{B}_{i.E}\hfill \\ {}\hfill {c}_i=c+{c}_{MOC}+{c}_{Year}+{c}_{Age}+{C}_{i.G}+{C}_{i.E}\hfill \end{array}, $$

where *k*, *b* and *c* are overall fixed parameter intercepts, *k*_*MOC/Year/Age*_, *b*_*MOC/Year/Age*_ and *c*_*MOC/Year/Age*_ are the fixed effects of each parameter for month of calving (MOC), year of milking (Year) and age (Age) of the cow at calving, *K*_*i.G*_, *B*_*i.G*_ and *C*_*i,G*_ are cow-specific polygenic random effects, and *K*_*i.E*_, *B*_*i.E*_ and *C*_*i,E*_ are cow-specific “environmental” random effects. These random components will have multivariate normal distributions such that:

5 $$ \left(\begin{array}{c}\hfill {K}_{i.G}\hfill \\ {}\hfill {B}_{i.G}\hfill \\ {}\hfill {C}_{i.G}\hfill \end{array}\right)\sim N\left(\left(\begin{array}{c}\hfill 0\hfill \\ {}\hfill 0\hfill \\ {}\hfill 0\hfill \end{array}\right),\left(\begin{array}{ccc}\hfill {\sigma}_{K.G}^2\hfill & \hfill {\sigma}_{KB.G}\hfill & \hfill {\sigma}_{KC.G}\hfill \\ {}\hfill {\sigma}_{KB.G}\hfill & \hfill {\sigma}_{B.G}^2\hfill & \hfill {\sigma}_{BC.G}\hfill \\ {}\hfill {\sigma}_{KC.G}\hfill & \hfill {\sigma}_{BC.G}\hfill & \hfill {\sigma}_{C.G}^2\hfill \end{array}\right)\right), $$

and

6 $$ \left(\begin{array}{c}\hfill {K}_{i.E}\hfill \\ {}\hfill {B}_{i.E}\hfill \\ {}\hfill {C}_{i.E}\hfill \end{array}\right)\sim N\left(\left(\begin{array}{c}\hfill 0\hfill \\ {}\hfill 0\hfill \\ {}\hfill 0\hfill \end{array}\right),\left(\begin{array}{ccc}\hfill {\sigma}_{K.E}^2\hfill & \hfill {\sigma}_{KB.E}\hfill & \hfill {\sigma}_{KC.E}\hfill \\ {}\hfill {\sigma}_{KB.E}\hfill & \hfill {\sigma}_{B.E}^2\hfill & \hfill {\sigma}_{BC.E}\hfill \\ {}\hfill {\sigma}_{KC.E}\hfill & \hfill {\sigma}_{BC.E}\hfill & \hfill {\sigma}_{C.E}^2\hfill \end{array}\right)\right), $$

where *i* indexes the cow, with *i* = 1, 2, …, *n* and *n* is the number of cows in the dataset.

First, considering the polygenic terms (*K*_*i.G*_, *B*_*i.G*_ and *C*_*i,G*_), it will be assumed that **Κ**_*G*_ = (*K*_1. *G*_, *K*_2. *G*_, …, *K*_*n. G*_)′, **Β**_*G*_ = (*B*_1. *G*_, *B*_2. *G*_, …, *B*_*n. G*_)′ and **C**_*G*_ = (*C*_1. *G*_, *C*_2. *G*_, …, *C*_*n. G*_)′ have the following distributions: ${\mathbf{K}}_G\sim N\left(\mathbf{0},{\sigma}_{K.G}^2\mathbf{Z}\mathbf{A}{\mathbf{Z}}^{\mathbf{\prime}}\right)$, ${\mathbf{B}}_G\sim N\left(\mathbf{0},{\sigma}_{B.G}^2\mathbf{Z}\mathbf{A}{\mathbf{Z}}^{\mathbf{\prime}}\right)$ and ${\mathbf{C}}_G\sim N\left(\mathbf{0},{\sigma}_{C.G}^2\mathbf{Z}\mathbf{A}{\mathbf{Z}}^{\mathbf{\prime}}\right)$, where **Z** links the “phenotypes” (*k*_*i*_, *b*_*i*_ or *c*_*i*_) to the animal pedigree records, and **A** is the numerator relationship matrix. In the current situation, it was assumed that only one lactation is simulated per cow, so **Z** = **I**_*n*_. It is also assumed that the “environmental” components **Κ**_*E*_ = (*K*_1. *E*_, *K*_2. *E*_, …, *K*_*n. E*_)′, **Β**_*E*_ = (*B*_1. *E*_, *B*_2. *E*_, …, *B*_*n. E*_)′ and **C**_*E*_ = (*C*_1. *E*_, *C*_2. *E*_, …, *C*_*n. E*_)′ are all independent and their covariances are assumed to be 0, i.e. ${\mathbf{K}}_E\sim N\left(\mathbf{0},{\sigma}_{K.E}^2{\mathbf{I}}_n\right)$, ${\mathbf{B}}_E\sim N\left(\mathbf{0},{\sigma}_{B.E}^2{\mathbf{I}}_n\right)$ and ${\mathbf{C}}_E\sim N\left(\mathbf{0},{\sigma}_{C.E}^2{\mathbf{I}}_n\right)$.

Putting these together, equation (5) yields:

7 $$ \left(\begin{array}{c}\hfill {\mathbf{K}}_G\hfill \\ {}\hfill {\mathbf{B}}_G\hfill \\ {}\hfill {\mathbf{C}}_G\hfill \end{array}\right)\sim N\left(\left(\begin{array}{c}\hfill {\mathbf{0}}_n\hfill \\ {}\hfill {\mathbf{0}}_n\hfill \\ {}\hfill {\mathbf{0}}_n\hfill \end{array}\right),\left(\begin{array}{ccc}\hfill {\sigma}_{K.G}^2\hfill & \hfill {\sigma}_{KB.G}\hfill & \hfill {\sigma}_{KC.G}\hfill \\ {}\hfill {\sigma}_{KB.G}\hfill & \hfill {\sigma}_{B.G}^2\hfill & \hfill {\sigma}_{BC.G}\hfill \\ {}\hfill {\sigma}_{KC.G}\hfill & \hfill {\sigma}_{BC.G}\hfill & \hfill {\sigma}_{C.G}^2\hfill \end{array}\right)\otimes \mathbf{A}\right) $$

(assuming **Z** = **I**_*n*_), and equation (6) yields

8 $$ \left(\begin{array}{c}\hfill {\mathbf{K}}_E\hfill \\ {}\hfill {\mathbf{B}}_E\hfill \\ {}\hfill {\mathbf{C}}_E\hfill \end{array}\right)\sim N\left(\left(\begin{array}{c}\hfill {\mathbf{0}}_n\hfill \\ {}\hfill {\mathbf{0}}_n\hfill \\ {}\hfill {\mathbf{0}}_n\hfill \end{array}\right),\left(\begin{array}{ccc}\hfill {\sigma}_{K.E}^2\hfill & \hfill {\sigma}_{KB.E}\hfill & \hfill {\sigma}_{KC.E}\hfill \\ {}\hfill {\sigma}_{KB.E}\hfill & \hfill {\sigma}_{B.E}^2\hfill & \hfill {\sigma}_{BC.E}\hfill \\ {}\hfill {\sigma}_{KC.E}\hfill & \hfill {\sigma}_{BC.E}\hfill & \hfill {\sigma}_{C.E}^2\hfill \end{array}\right)\otimes {\mathbf{I}}_n\right). $$

### Simulated data

From the initial nlme() model output that fitted the Wood model to the raw Sahiwal lactation records, estimates of the fixed effects of *k, b,* and *c* and the variance and covariance matrix of the combined random effects (*K*_*i.G*_ *+ K*_*i.E*_, *B*_*i.G*_ *+ B*_*i.E*_ and *C*_*i.G*_ *+ C*_*i.E*_) were obtained. This variance-covariance matrix was split up to resemble the separate random cow-effects (*K*_*i.G*_, *B*_*i.G*_ and *C*_*i.G*_) and the random “environmental” effects (*K*_*i.E*_, *B*_*i.E*_ and *C*_*i.E*_), such that simulated lactation curves yielded realistic curves. For the purposes of the simulation, non-zero covariances were used in equation (7) but all covariances in equation (8) were set equal to 0 (see Additional file 1). These two variance-covariance matrices and the relationship matrix (**A**, based on the LPRI pedigree) were used with the rmvnorm() [31] function of R Version 3.0.2 [29] to generate random effects drawn from multivariate normal distributions (equations (7) and (8)) for a simulated population. These were then added to the estimates of fixed effects parameters (from the Sahiwal lactation data) to yield realistic simulated values for *k*_*i*_*, b*_*i*_ and *c*_*i*_ according to equation (4).

**Figure 1:**
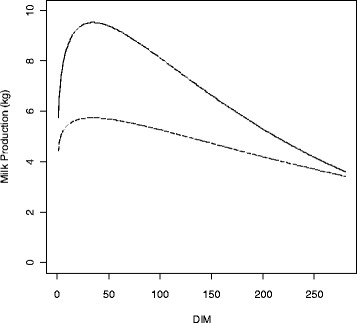
**The ‘average’ shape of simulated lactation curves using Wood’s model based on Pakistani Sahiwal data.** One population of animals was simulated with an average peak and persistent tail (APHP; dashed line ---), which represented 4-year old animals. The other population had a higher peak and less persistent tail (HPLP; solid line ―) to simulate 9-year old animals.

The outcomes of this simulation were values for *k*_*i*_, *b*_*i*_ and *c*_*i*_ for a population of Sahiwal cattle (where *n* = 464) that calved in January 2006 at 4 years of age. The resulting lactation curves had a general shape that had an average peak of production and high persistency (APHP; a slowly declining curve). A second set of data was simulated in which only the fixed effect of age was changed to 9 years to generate lactation curves that on average had a higher peak and a less persistent tail (HPLP). The fixed effects of 4 and 9 years of age were selected for the simulation since they yielded lactation curves that were quite different in shape, yet still typical of Sahiwal cows. Plots of the average simulated lactation curves that highlight differences between these fixed effects are in Figure 1.

To ensure that the simulated datasets were realistic to the Pakistani situation, a random error term ε_*it*_ was added to each simulated day of milking (according to equation (3)) to take into account the important environmental variation that occurs in milk production due to daily variation in nutrition, management and other factors. Note that the random errors were taken as independent, although in reality they may be serially correlated with an autoregressive error structure.

To calculate the true simulated lactation yield (*Y*) for animal *i* within the herd, the yield on each DIM (*t* = 1, 2, 3,…, 280) was summed according to the following equation;

9 $$ {Y}_{i,\mathrm{TRUE}}={\displaystyle {\sum}_{i=1}^{280} \exp \left({k}_i+{b}_i \ln t-{c}_it\right)+{\varepsilon}_{it}.} $$

This value *Y*_*i,*TRUE_ was calculated for each animal and is considered the ‘true’ lactation yield from the simulations. This was done separately for the simulated datasets for which animals were 4 (APHP) and 9 (HPLP) years old. An assumed lactation length of 280 days was used here since the average lactation length in Sahiwal cattle in Pakistan is 235 days [10] and only a small proportion of animals produce milk for more than 305 days [32].

### Test-day sampling regimes tested

Standard lactation yield estimation methods use the TIM based on TD records collected at equally spaced weekly, monthly and quarterly intervals. By sub-setting the simulated data to represent these recording regimes, lactation yields were calculated (*Y*_*i*,TIM,WKL_, *Y*_*i*,TIM,MON_, *Y*_*i*,TIM,QTR_) and used for comparison with other TDSR and estimations using Wood’s model.

**Figure 2:**
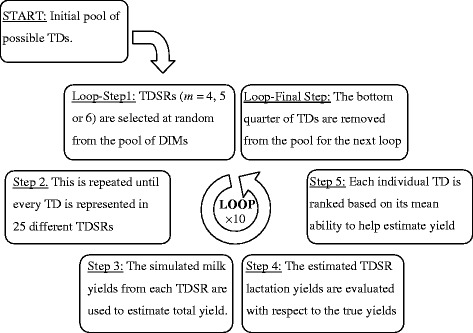
**Flow diagram describing the process of the random test-day sampling regime (TDSR) selection.** This diagram outlines the method in which the pool of days in milk (DIM) to select *m* = 4, 5, or 6 test-days (TD) from is refined following each loop.

The main aim of this study was to investigate the possibility of using fewer (*m*) TD per lactation (where *m* is equal to 4, 5 or 6), strategically timed throughout lactation to estimate lactation yield. In order to determine the ‘ideal’ TDSR, numerous TD combinations were tested and compared. The number of possible TDSR within a single lactation is very large. For example, from a lactation of 280 DIM, if each combination of five (*m* = 5) randomly selected TD was tested, there would be over 1.6 × 10^12^ possible combinations. Since it is unrealistic to compare each of these possible TDSR, a method was developed to refine the selection process of the TDSR to be tested based on previous results. A diagram of this process is in Figure 2, and details are given below.

The process of TDSR comparison was carried out over a number of loops by selecting combinations of *m* (= 4, 5 or 6) TD from the pool of DIM. In the first loop, the pool of TD included all DIM (1,…, 280). In subsequent loops, the pool of DIM was reduced by one quarter based on the results from the previous loop (see Figure 2). The steps to compare TD within each loop were as follows:

- Step 1: Select *m* TD at random from the pool without replacement. This will make up one TDSR for comparison (TDSR_*j*_). This was repeated until every TD from the pool was represented at least once in a selected TDSR_*j*_.
- Step 2: Repeat ‘Step 1’ until each TD in the pool is represented in at least 25 different TDSR_*j*_, to ensure that there are enough repeated measurements to justify valid comparisons when TD are evaluated. Fewer repeats could have been used (for example 5), but for the purposes of this study, 25 were used to allow for greater certainty in the assessment of the contribution of each DIM to the lactation yield estimate.
- Step 3: For each randomly selected TDSR_*j*_, the corresponding TD yields in the simulated population were used to estimate lactation yields for each cow. This was done by fitting Wood’s lactation curve model using the nonlinear mixed effects model by the nlme() function of R Version 3.0.2 [29]. The fitting process and lactation yield estimation followed the processes outlined by Raadsma et al. [22]. This resulted in a lactation yield estimate for each cow, using Wood’s model based on TD records from the TDSR_*j*_ (*Y*_*i*,WOOD,TDSRj_).
- Step 4: For every TDSR_*j*_, the mean square error of prediction (MSEP) was calculated using the following formula:
  10 $$ {\mathrm{MSEP}}_{\mathrm{WOOD},{\mathrm{TDSR}}_j}=\frac{1}{n}{\displaystyle {\sum}_{i=1}^n{\left({Y}_{i,\mathrm{TRUE}}-{Y}_{i,\mathrm{WOOD},{\mathrm{TDSR}}_j}\right)}^2}, $$
  where *n* is the number of simulated lactation yields.
- Step 5: The 25 different TDSR_*j*_ that contained each TD were averaged to yield a single average MSEP for each TD. Using this value, each TD from the pool was ranked from the highest MSEP (most inaccurate) to the lowest.
- Step 6: One-quarter of the TD with the most inaccurate average MSEP were removed from the pool. This reduced the pool of TD for the next loop to theoretically include TD that on average allow for more accurate estimation of lactation yield.

This loop process can theoretically continue until the number of TD in the pool is less than the number of TD (*m*) per TDSR. It was expected that the MSEP would initially reduce as the size of the pool decreased but then either reach a plateau or increase again, i.e. decrease in accuracy. For the purposes of this study, the looping process was repeated ten times, when the pool had only 20 DIM remaining.

### Comparisons

The methods used to estimate lactation were compared in three ways: (1) MSEP between true and estimated lactation yield, (2) comparison of EBV based on true lactation yield and estimated lactation yield and (3) number of sires that could be theoretically tested for a given number of test-day recordings. More detailed descriptions of these comparisons are given below.

First, the simulated lactation yields were compared directly with the various estimates of lactation yield using the MSEP. Simulated lactation yields (*Y*_*i,*TRUE_) were compared with the estimates obtained with methods based on fewer TD records. This included the estimation methods already described (${Y}_{i,\mathrm{T}\mathrm{I}\mathrm{M},\mathrm{W}\mathrm{K}\mathrm{L}},\ {Y}_{i,\mathrm{T}\mathrm{I}\mathrm{M},\mathrm{MON}},\ {Y}_{i,\mathrm{T}\mathrm{I}\mathrm{M},\mathrm{Q}\mathrm{T}\mathrm{R}},\ {Y}_{i,\mathrm{W}\mathrm{OOD},{\mathrm{TDSR}}_j}$) and additionally, the TIM at the different TDSR_*j*_ (${Y}_{i,\mathrm{T}\mathrm{I}\mathrm{M},{\mathrm{TDSR}}_j}$) and the Wood method at the equally spaced sampling regimes (*Y*_*i*,WOOD,WKL_, *Y*_*i*,WOOD,MON_, *Y*_*i*,WOOD,QTR_). Using the estimates of lactation yield, MSEP were calculated (similarly to that described in equation (10)) and used to compare the accuracy of the different lactation yield estimation methods.

Second, the predicted EBV from each method were compared with the ‘true’ EBV based on the simulated data. EBV for each cow were calculated based on the lactation yield estimates using the TIM (*EBV*_*i*,TIM,WKL_, *EBV*_*i*,TIM,MON_, *EBV*_*i*,TIM,QTR_, $EB{V}_{i,\mathrm{T}\mathrm{I}\mathrm{M},{\mathrm{TDSR}}_j}$) and the Wood method (*EBV*_*i*,WOOD,WKL_, *EBV*_*i*,WOOD,MON_, *EBV*_*i*,WOOD,QTR_, $EB{V}_{i,\mathrm{WOOD},{\mathrm{TDSR}}_j}$). These EBV were calculated in ASReml-R Discovery Edition 1.0 [33], using the **A** matrix based on the LPRI pedigree data. The EBV based on the simulated data using the true lactation yields (*Y*_*i,*TRUE_) were also calculated (EBV_*i,*TRUE_). EBV of the different lactation yield estimation methods were compared by using the number of cows in the top 100 EBV that corresponded with the ‘true’ top 100 EBV (Top100). This enabled an assessment of the similarities between methods to estimate lactation yield in terms of the cows that would be theoretically selected.

Lastly, the number of sires that could theoretically be progeny-tested was used as a comparison to determine which method could use allocated resources most efficiently. According to the method outlined by Duclos et al. [27], the theoretical reliability (*R*) of a sire can be calculated as:

11 $$ R=\frac{d{h}^2}{4+\left(d-1\right){h}^2+4\left({\sigma}_{\varDelta}^2/{\sigma}_P^2\right)}, $$

where *h*^*2*^ is the heritability of *Y*_*i,*TRUE_, *d* is the number of daughters and ${\sigma}_P^2$ is the phenotypic variance of *Y*_*i,*TRUE_. The ${\sigma}_{\varDelta}^2$ represents the increase in the residual variance due to the TDSR recording protocol and can be calculated as the variance of the differences between the estimated yield under the TDSR recording protocol, *Y*_*i*,ESTIMATE_, and yield under a full recording protocol, *Y*_*i*,TRUE_,

12 $$ {\sigma}_{\Delta}^2=\mathrm{V}\mathrm{a}\mathrm{r}\left({Y}_{i,\mathrm{ESTIMATE}}-{Y}_{i,\mathrm{TRUE}}\right). $$

Using these equations and simple algebra, an expression can be developed to determine the theoretical number of daughters necessary to prove a sire with a specific reliability level. Then, the number of sires that could possibly be proven given a limited number of resources can be calculated and compared according to the method used to estimate lactation yield.

In Pakistan, the current Sahiwal progeny testing system (not including government farms) records data monthly from approximately 30 private farms, which we assumed, had 25 milking animals each. This means that 7500 (30 × 25 × 10) TD records can be collected within a given lactation. Thus, the number of possible proven sires is equal to:

13 $$ \frac{7500}{m\times d}, $$

where *m* is the number of TD recorded per lactation and *d* is the number of daughters necessary (calculated using equation (11)) to prove a sire with a given reliability (*R*) and ${\sigma}_{\varDelta}^2$ based on the method of lactation yield estimation.

## Results

TDSR selection and comparison were done separately for the two lactation curve shapes (APHP and HPLP) and for *m* recorded TD (where *m* is equal to 4, 5 or 6) within a lactation. This allows for comparison of the key outcomes and practical applications of these results based on both shape of the lactation curve and the number of TD recorded.

### Test-day sampling regimes tested

**Figure 3:**
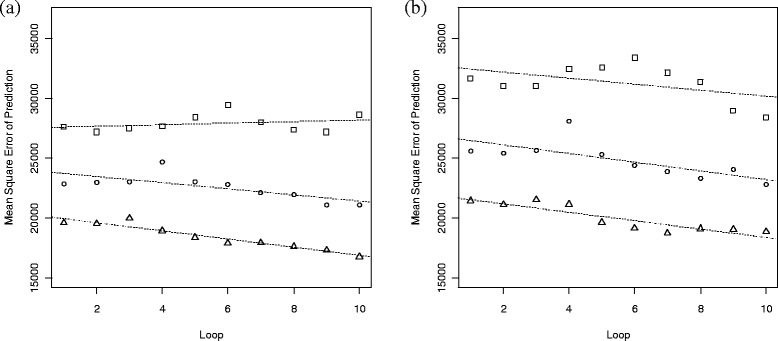
**Plots of the trend of the median mean square error of prediction with each loop.** Plot **(a)** shows the trend for the simulated lactations with an average peak and high persistency, APHP and **(b)** for the simulated lactations with a high peak and low persistency, HPLP; the symbols represent the number (*m*) of TD per lactation used to estimate the lactation yield, where □ is for *m* = 4, o for *m* = 5 and Δ for *m* = 6; the regression lines show the trend of median MSEP values from loop 1 through to loop 10.

Selection of the TDSR to be compared within this study was done using a process that removed DIM with each loop in order to reduce the number of possible combinations of TDSR while maintaining the DIM that contributed to accurate estimates of lactation yield using Wood’s model. We can test the efficacy of this process by examining the trend of the median MSEP of all the TDSR tested within each loop (Figure 3). These plots show that, in general, the median MSEP decreased with each subsequent loop. However, this was not the case when the shape of the curves were APHP and *m* = 4 TD, for which a small increase in the median MSEP was observed (Figure 3a). The general downward trend of the median MSEP with each loop shows that, overall, the accuracy of the TDSR improved with an increasing number of loops. However, this does not necessarily mean that the TDSR with the lowest MSEP will be in the last loop because the TDSR were chosen at random from the remaining pool of TD in each loop. Thus, it is possible to randomly choose the TDSR with the lowest MSEP in the earlier loops, although this is not very likely because there are more DIM to choose from.

### Comparison of lactation yield estimates

**Figure 4:**
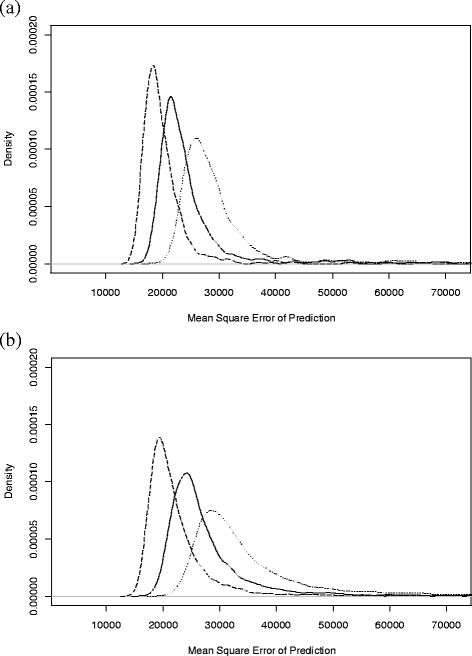
**Probability density plots of the mean square error of prediction from Wood model lactation estimates.** The probability density plots are shown separately for random selections of test-day (TD) sampling regimes of 4, 5 and 6 TD per lactation; plot **(a)** shows the trend for the simulated lactations with an average peak and high persistency (APHP) and **(b)** for the simulated lactations with a high peak and low persistency (HPLP); the line type represents the number (*m*) of TD per lactation used to estimate the lactation yield, where dotted (•••) is for *m* = 4, solid (―) for *m* = 5 and dashed (−−−) for *m* = 6.

The key question in this study was how lactation yields estimated with Wood’s model [12] using fewer TD records compare with estimates from the recommended TIM method [9]. The plots in Figure 4 show the distribution of the MSEP values for the TDSR using four, five or six TD to estimate lactation yield. Lower values of MSEP indicate more accurate estimates of lactation yield.

The TDSR method based on six TD per lactation resulted in comparable estimates of lactation yield as the TIM with monthly records, which had MSEP values of 12 385 (APHP) and 13 587 (HPLP), as shown in Table 1. This is indicated in Figure 4a and 4b by the location of the left-tail of the dashed MSEP curve (*m* = 6), which indicates that a small proportion of the TDSR tested had MSEP values that were less than or equal to the MSEP for the TIM monthly estimates. Similarly, the position of all three probability density plots show that the use of Wood’s model with *m* TD (where *m* = 4, 5 or 6) to estimate lactation yield produced more accurate estimates than the TIM with quarterly records (MSEP values; 43 621 for APHP and 71 631 for HPLP) but less accurate estimates than TIM with weekly records (MSEP values; 2936 for APHP and 3112 for HPLP).

**Table 1:** **Comparison of different estimation methods and sampling protocols**

**Sampling protocol**	**Average peak and persistent tail**		**High peak and less persistent tail**	
	**Top 100**_**Med**_	**MSPE**_**Med**_	**Top 100**_**Med**_	**MSPE**_**Med**_
Test-interval method				
Weekly^†^	96	2936	96	3112
Monthly^†^	85	12385	88	13587
Quarterly^†^	73	43621	77	71631
6 Test-days/lact^‡^	82	26829	87	32229
5 Test-days/lact^‡^	81	30649	86	35479
4 Test-days/lact^‡^	81	35767	85	47048
Wood model estimation				
Weekly^†^	96	2613	96	2945
Monthly^†^	87	10530	90	12098
Quarterly^†^	73	31334	82	34508
6 Test-days/lact^‡^	82	18511	87	20666
5 Test-days/lact^‡^	81	21654	85	24964
4 Test-days/lact^‡^	81	25306	84	30324

^†^For the equally spaced estimation methods (weekly, monthly, quarterly) only one regime was tested and this value is reported here; ^‡^for the different TDSR (*m* = 4, 5 and 6 test-days/lactation), the values presented here are the median top100 and MSPE values for all the TDSR tested that have at least 80 animals in common in the top 100 compared to the ‘true’ EBV.

### Comparison of estimated breeding values

**Figure 5:**
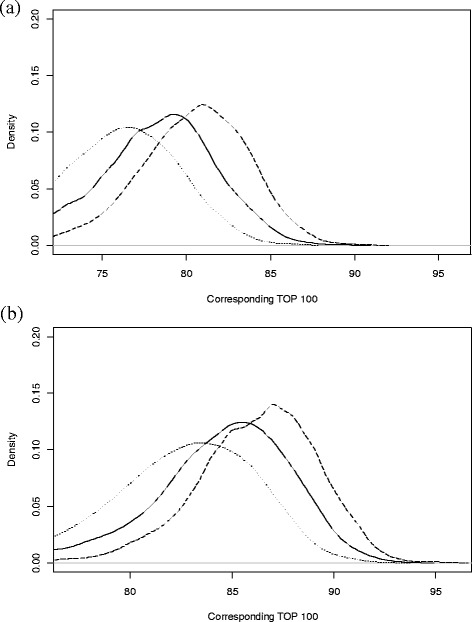
**Probability density plots of the corresponding top 100 ranked animals between estimated and simulated data.** Plot **(a)** shows the trend for the simulated lactations with an average peak and high persistency (APHP) and **(b)** for the simulated lactations with a high peak and low persistency (HPLP); the line type represents the number (*m*) of TD per lactation used to estimate the lactation yield, where dotted (•••) is for *m* = 4, solid (―) for *m* = 5 and dashed (−−-) for *m* = 6.

The accuracy of lactation yield estimates, measured by the MSEP, is an important parameter to compare different methods of estimation. However, an easier to apply measure when dealing with animal selection is to determine the correspondence of the rankings of EBV calculated using the alternate methods compared with the ‘true’ EBV rankings based on the simulated lactation yields. Figure 5 shows the distribution of the number of cows in the top 100 EBV that corresponded with the ‘true’ top 100 EBV. In this figure, values closer to 100 are considered more accurate.

In both Figure 5a and 5b, the probability density plots show that there was a large proportion of TDSR that had greater Top100 values than the estimates with the TIM based on quarterly records (Table 1; APHP - 73, HPLP - 77) and to a lesser extent the monthly records (Table 1; APHP - 85, HPLP - 88). As the number (*m*) of TD recorded per lactation increased, the proportion of TDSR that had a greater correspondence with the ‘true’ EBV rankings also increased compared to estimates obtained based on monthly records. This was even truer when the graph shape showed a rapid decline (HPLP) as compared to a slow decline (APHP). The median values for the probability density plots are in Figure 5 and Table 1. Results in Table 1 show that, as the frequency of recording increased, the median MSEP decreased. Similarly, as the median MSEP decreased the correspondence between the EBV rankings and ‘true’ EBV rankings increased. The values of the Top100 corresponding animals show that using four, five or six TD, strategically timed throughout lactation, allowed for the selection of animals that aligned better with the ‘true’ EBV than the ‘TIM Quarterly’ regime and in some cases the ‘TIM Monthly’ regime.

Another measure that can be used to compare methods is the correlation of the resulting EBV with the ‘true’ EBV. For the APHP curves using four, five or six TD, these correlations were equal to 0.882, 0.905 and 0.923, respectively. For the HPLP curves, correlations were equal to 0.931, 0.947 and 0.959. These values concur with the trends seen in the Top100 values but give a more direct indication of the potential amount of genetic gain lost because of using fewer TD per lactation.

### Comparisons of sires tested

By applying selection index theory, the EBV results can be further extrapolated for application within a progeny-testing situation. Table 2 shows the bias and *σ*_*Δ*_ of the different methods to estimate lactation yield. The *σ*_*Δ*_ is the standard deviation of the deviations between true and estimated lactation yields using equation (12), whereas the bias is the mean of the differences between true and estimated lactation yields, (*Y*_*i*,ESTIMATE_ − *Y*_*i*,TRUE_). The resulting values of ${\sigma}_{\varDelta}^2$ were used in equation (11) to calculate the number of daughters necessary to prove a sire with a reliability of 50%. Subsequently, assuming that a given amount of TD recording resources was available, the number of possible sires that could be tested was determined using equation (13). Results show that accuracy of the lactation yield estimate tended to increase as the number of TD per lactation increased. Hence, with the more accurate methods to estimate lactation yield, fewer daughters were required to prove a sire with a given reliability. However, the results also indicate that, with limited resources, the more frequent TD sampling reduced the number of sires that could be proven.

**Table 2:** **Comparisons between estimation methods and sampling protocols based on the number of possible sires tested**

**Sampling protocol**	**Average peak and persistent tail**			**High peak and less persistent tail**		
	**Average bias (±σ**_**Δ**_ **)** ^**†**^	**Number daughters**	**Possible sires tested**	**Average bias (±σ**_**Δ**_ **)** ^**†**^	**Number daughters**	**Possible sires tested**
Weekly	−9 (±53)	16.3	11.2	−5 (±56)	15.3	11.9
Monthly	−18 (±110)	19.3	35.4	−29 (±113)	16.8	40.7
Quarterly	−64 (±199)	28.0	66.9	−177 (±201)	20.9	89.6
6 Test-days/lact	5 (±163)	23.9	52.4	61 (±169)	19.2	65.3
5 Test-days/lact	7 (±174)	25.1	59.8	46 (±182)	19.8	75.8
4 Test-days/lact	10 (±188)	26.7	70.2	76 (±202)	21.0	89.3
Weekly	2 (±51)	16.3	11.2	7 (±54)	15.3	12.0
Monthly	14 (±102)	18.7	36.4	21 (±108)	16.6	41.1
Quarterly	33 (±174)	25.1	74.8	31 (±183)	19.9	94.3
6 Test-days/lact	20 (±134)	21.1	59.1	19 (±142)	17.9	70.0
5 Test-days/lact	23 (±145)	22.1	67.9	21 (±156)	18.5	81.1
4 Test-days/lact	27 (±156)	23.1	81.1	25 (±171)	19.2	97.6

The values reported in this table are the median values calculated from the TDSR tested that have at least 80 animals in common in the top 100 compared to the ‘true’ EBV; ^†^the ‘Average Bias’ of each method is presented here with its residual standard deviation (*σ*_*Δ*_, calculated using equation 12); the ‘Number Daughters’ reports the theoretical number of daughters that would be required given the *σ*_*Δ*_ of the estimation method to prove one sire with a reliability of 50%, and a heritability if 0.2 (calculated using equation 11); the ‘Possible Sires Tested’ shows the predicted number of sires according to equation 13 that could theoretically be proven (with a reliability of 50%) given the required number of daughters.

### ‘Ideal’ test-day sampling regime

**Figure 6:**
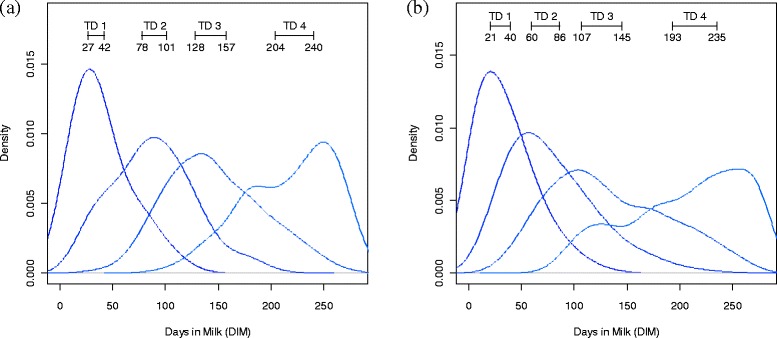
**Distributions of recommended sampling protocols with four TD per lactation.** These plots show distributions of the sampling protocols for the TDSR with four TD per lactation and have over 80 of their top 100 ranked animals in common with the ‘true’ EBV ranks; plot **(a)** shows the distributions for the average peak high persistency simulations and **(b)** shows those for the high peak, low persistence simulations; the values in brackets on the plots for each of the four TD show the range of the middle 25% of DIM for each distribution.

Using the MSEP values from the simulation study, an ideal sampling regime for collecting TD records can be recommended. The accuracy of lactation yield estimates and subsequent EBV predictions with the TDSR evaluated varied greatly. To develop possible recommendations for sampling regimes, a criterion was established to subset the TDSR into those that were accurate enough for selection and those that were not. For this study, the criterion was set such that the estimation method had to have over 80 animals in the top 100 ranked EBV which corresponded with the ‘true’ EBV ranks. Of all the TDSR tested with *m* = 4 TD within a lactation, 18.1% of the APHP and 63.9% HPLP shaped curves met this criteria. Figure 6 shows the distribution of the sampling days (one to four) for the TDSR that met these criteria. Similar figures could be produced for *m* = 5 and *m* = 6 as well, but for the purposes of the discussion we will focus on *m* = 4. Figure 6 shows that it was necessary to have the first TD early in lactation, around the peak of lactation (where the average peak occurs at 34 DIM for APHP and 35 DIM for HPLP).

Using Kolmogorov-Smirnov goodness of fit tests [34], the distributions from Figure 6 were shown to differ significantly from the distributions of *m* = 4 order statistics from a uniform *U*(0,280) distribution (all *P* <10^−11^). Therefore, the results from this study suggest that strategically timing TD sampling throughout the lactation yielded more accurate estimates of lactation yield than random (uniform) sampling.

## Discussion

This study investigated methods to estimate lactation yield more accurately in a developing dairy sector where resources for data collection and progeny-testing are limited. The focus was to investigate estimation methods using fewer, yet more strategic, TD sampling regimes and to propose a methodology to progeny-test bulls for milk production when milk recording is limited. The two key comparisons were: (1) the TIM with Wood’s lactation curve model and (2) TD recording regimes with equal intervals (weekly, monthly and quarterly) with TD recording regimes with four, five or six records per lactation more strategically placed throughout lactation.

The MSEP_Med_ results (Table 1) show that in all cases, the TIM had higher MSEP_Med_ than the corresponding recording regime using the Wood model with differences ranging from 5% (HPLP-Weekly) to more than double (for HPLP-Quarterly). This suggests that estimating lactation yield with the Wood model is more accurate than with the TIM. Therefore, if genetic gain was predicted based on the accuracy of EBV from this analysis, using the Wood model would be superior. However, the differences between the estimation methods are not as large based on the corresponding Top100_Med_ values, which were either the same or differed by only one or two animals ranked in the top 100, with a maximum difference of 5 for HPLP-Quarterly. This suggests that the TIM and Wood model methods ranked the animals similarly for selection purposes and hence genetic progress would be essentially the same, regardless of the method.

Although the selection outcomes of the TIM and Wood model estimation methods may be the same, if we extrapolate this information further, the difference in the accuracy of the estimation will have an effect in the long-term. Based on theoretical calculations, the Wood model estimation method can prove more sires (Table 2) than the TIM with the same TD sampling regime. Therefore, for a given TD sampling regime, we would expect the Wood model method to yield more accurate lactation yield estimates and hence use the available resources more efficiently. Nonetheless, it should be noted that nonlinear models other than the Wood model may also be appropriate. The main consideration here was to adopt a model with relatively few parameters, bearing in mind the relatively small number of observations (*m*) per lactation.

With regard to the number of TD recorded per lactation, the results in Table 2 show that as the number of records within lactation decreased, the residual standard deviation (*σ*_*Δ*_) of the bias in the estimation method increased consistent with other studies [26,28]. Using this *σ*_*Δ*_ value and equations (11) and (13), we can determine the theoretical number of daughters required to prove a sire to attain a reliability (*R*) of 50% (Table 2). These results show that as *σ*_*Δ*_ increases, more daughters are required to prove a sire with a given reliability. Despite this, recording fewer TD per lactation provides an opportunity to record more cows with the same resources allocated to the progeny-testing system (Table 2). Thus, recording regimes with fewer TD per lactation and using the Wood model method to estimate lactation yield are the most efficient in terms of use of the resources. The implications of this to the overall outcome of the progeny-testing program is a greater pool of progeny-tested sires to select from, which means that genetic gain can be increased by increasing selection intensity. Duclos et al. [27], using similar calculations, also concluded that more animals can be tested by using fewer TD records in a lactation, without affecting the reliability of the bulls’ EBV.

The novelty of this study lies in the testing of different strategically placed TDSR, with the aim of finding TDSR that provide the most accurate lactation yield estimates and the highest accuracy to select animals. The purpose of the loops and selection process for the TDSR was to find these ‘ideal’ recording regimes. The results of this study show that more DIM are required in the earlier portion of the lactation than in the latter portion. This agrees with the idea discussed in previous research that suggests the earlier portion of lactation is more difficult to model than the later portion [27,28]. Looking at all the TDSR tested and their lactation yield estimates, Figure 3 shows the median MSEP from each loop. The general trend of both these plots (for APHP and HPLP) is that the median MSEP decreased with each loop. This suggests that as the loop process continues, it is testing TDSR that allow for more accurate lactation yield estimates and hence is more likely to find the ‘ideal’ TDSR.

Despite the positive outcomes of the loop process and the selection of superior TDSR, a number of issues must be considered. First, in reality, each cow has a different recording regime since not all cows will give birth at the same time. Furthermore, data collection occurs at different farms at different times. Therefore, even if one ‘ideal’ TDSR was found, it would be difficult to implement that precise recording regimen. Therefore, a more realistic outcome of this research is to develop recommendations about possible ranges of TD sampling times that yield ‘good’ estimates of lactation. Figure 6 shows the distribution of the four TD of the TDSR tested with over 80 animals in the top 100 ranked EBV which corresponded with the ‘true’ EBV ranks. The distribution and intersection of these curves indicate that there is a range for each TD, which, if followed, allows for adequate lactation yield estimation. The ranges shown in Figure 6 suggest that, although the frequency of TD in the later stages of lactation was not as high, it is important to have TD both pre-peak and post-peak lactation. Other studies have reported similar results, which suggest that the first TD should be recorded early in lactation [26] and post-peak sampling is important in the estimation procedure [28]. There is evidence to suggest that unequal intervals between TD lead to more biased estimates than equidistant records [27]. However, this study has completed a more thorough and direct comparison of these differences and found that unequal TD sampling intervals strategically placed throughout the lactation can provide less biased lactation yield estimates.

The simulation process used in this study assumed a lactation length of 280 days and an approximate heritability for cumulative milk yield of 0.2 (see Additional file 1). These values were used to ensure that the simulated lactations were similar to those of the Sahiwal population in Pakistan. If a longer lactation length (for example, 305 days) was used, the only difference, if any, could be slight changes in the recommended timeframes from which one should take TD samples (Figure 6). This is because, although the overall ‘length’ of the lactation would be longer, the key characteristics of the curve (peak, inflection) would not change and so the ‘ideal’ TD sampling times, which presumably revolve around these characteristics, would also not change. With regard to heritability, if for example, a higher heritability was used, partitioning of variation in the raw estimates of the parameters of the Wood model between polygenic random effects and cow-specific “environmental” random effects would be different (see Additional file 1). This would lead to simulated lactation curves that would be more similar than the lactation curves simulated in this study. The implications of this could lead to lower MSPE values in all TDSR, but the general comparative differences and recommendations would ultimately be expected to be the same.

It can be argued that the use of TDM or daily milk yields would be beneficial in developing progeny-testing systems since it would allow for the inclusion of unfinished lactations and handle the analysis of lactations with few records [25]. Several publications suggest that TDM can supersede selection based on even completed lactation yields [35,36] because with improved statistical methods, both environmental and genetic effects [4,37] are better accounted for and can yield more precise definitions of contemporary groups and stage of lactation [38-40]. However, for these methods to be effective, accurate estimates of genetic and phenotypic parameters are required [3,8] which are difficult to obtain in developing countries [3] because in many cases field recording is inefficient and poor [41]. Research on data from Pakistan shows that TDM could be used [42] but this was based on a limited dataset. In the future, as more TD data become available electronically, the use of a fixed regression TDM could be a viable option. Furthermore, if TD are strategically placed, as suggested in this study, it could aid in the estimation of the parameters that describe the lactation curve shape in the fixed regression TDM. However, due to the current level of recording and electronic data entry, this study did not consider a TDM suitable for the Pakistani situation and instead looked at various approaches for which lactation yield is first estimated and subsequently used for breeding value estimation.

## Conclusions

The results of this study show that using Wood’s model to estimate lactation yield is more accurate than the TIM, although selection outcomes in terms of the ranking of EBV were very similar. Results also show that using few TD records (say four, five or six TD within one lactation) that are more strategically placed throughout lactation can produce more accurate estimates of lactation yield than a quarterly recording regime and have the potential to be as accurate as a monthly recording regime. Lastly, although using fewer TD causes an increase in the residual standard deviation for the lactation yield estimate, they provide an opportunity to progeny-test more sires and thus for a more efficient use of the allocated resources. Although this study was based on data from Sahiwal cattle in Pakistan, these recommendations can be applied to any dairy breed with similar lactation curve characteristics.

## Additional file

Additional file 1:
**Variance matrices used for simulation.** This file contains a description of the variance matrix values for the genetic and environmental effects used for the simulation of lactations in this study.
