# Supplementary material for: Strategic test-day recording regimes to estimate lactation yield in tropical dairy animals
Source: Genet Sel Evol. 2014 Dec 2;46(1):78. doi: 10.1186/s12711-014-0078-0 (PMC4248470; doi:10.1186/s12711-014-0078-0)
Supplement: Additional file 1: — Variance matrices used for simulation. This file contains a description of the variance matrix values for the genetic and environmental effects used for the simulation of lactations in this study. [file 12711_2014_78_MOESM1_ESM.pdf]

Additional file 1 – Variance matrices used for simulation

This file contains a description of the variance matrix values for the genetic and environmental effects used for the simulation of lactations in this study. The diagonal values in the matrices below sum to the variance estimates of the parameters of the Wood model based on the nlme() analysis of the Pakistan Sahiwal herd recorded data. The covariance estimates of the multivariate normal genetic effects were calculated based on the correlation from the same nlme() analysis. The covariance of the multivariate environmental effects was assumed to be zero. The proportions of variance were allocated to the diagonals of the genetic and environmental effects such that they simulated daily and total lactation yields that were realistic to the Pakistani Sahiwal population and had a heritability of approximately 0.2. A heritability of 0.2 is assumed here based on the findings of previous research on the Sahiwal cattle population in Pakistan [1, 2].

Multivariate normal genetic effects:

$$\begin{pmatrix} \mathbf{K}_G \\ \mathbf{B}_G \\ \mathbf{C}_G \end{pmatrix} \sim N\left(\begin{pmatrix} \mathbf{0}_n \\ \mathbf{0}_n \\ \mathbf{0}_n \end{pmatrix}, \begin{pmatrix} 5.755\times10^{-2} & & \\ -1.126\times10^{-2} & 6.389\times10^{-3} & \\ -2.535\times10^{-5} & 1.044\times10^{-4} & 2.726\times10^{-6} \end{pmatrix} \otimes \mathbf{A}\right)$$

Multivariate normal environmental effects:

$$\begin{pmatrix} \mathbf{K}_E \\ \mathbf{B}_E \\ \mathbf{C}_E \end{pmatrix} \sim N\left(\begin{pmatrix} \mathbf{0}_n \\ \mathbf{0}_n \\ \mathbf{0}_n \end{pmatrix}, \begin{pmatrix} 2.302\times10^{-2} & & \\ 0 & 2.556\times10^{-3} & \\ 0 & 0 & 1.818\times10^{-7} \end{pmatrix} \otimes \mathbf{I}_n\right)$$

Additional File References

1. Bajwa IR, Khan MS, Ahmad Z, Gondal KZ: **Genetic parameters of lactation milk yield as affected by lactation length adjustment procedures** in *7th World Congress on Genetics Applied to Livestock Production 19–23 August 2002, Montpellier, France* 2002, pp. 1-56.

2. Dahlin A, Khan UN, Zafar AH, Saleem M, Chaudhry MA, Philipsson J: **Genetic and environmental causes of variation in milk production traits of Sahiwal cattle in Pakistan**. *Animal Science*, **66** (1998), 307-318.
